# Navigating the Complication: Acute Mastoiditis Causing Cerebral Venous Thrombosis in an Adult

**DOI:** 10.7759/cureus.72863

**Published:** 2024-11-01

**Authors:** Kevin T Dao, Elias Inga Jaco, Edvard Davtyan, Matthias Park, Kasey Fox, Igor Garcia-Pacheco

**Affiliations:** 1 Internal Medicine, University of California, Los Angeles (UCLA) - Kern Medical, Bakersfield, USA; 2 Emergency Medicine, University of California, Los Angeles (UCLA) - Kern Medical, Bakersfield, USA; 3 Internal Medicine/Critical Care Medicine, University of California, Los Angeles (UCLA) - Kern Medical, Bakersfield, USA

**Keywords:** acute mastoiditis, adult, cerebral venous thrombosis, complications, hypercoagulable, otitis media, thrombophilia

## Abstract

Acute mastoiditis is an infection of the air cells in the mastoid and is primarily seen in the pediatric population. This disease usually occurs after patients develop otitis media, which can result in acute mastoiditis as a complication. Most patients usually present with generalized symptoms of an infection; however, in some instances, cerebral venous thrombosis can occur. Such a rare complication has been described in children, although in rare instances, it can happen in adults. In fact, only a few cases of acute mastoiditis resulting in cerebral venous thrombosis have been reported in adults, but usually, such patients have some form of underlying thrombophilia. However, in this case, a thrombophilia workup was unremarkable. Here, a patient presented to the emergency department and developed cerebral venous thrombosis secondary to acute mastoiditis without evidence of thrombophilia.

## Introduction

Acute mastoiditis is an infection of the air-filled spaces in the mastoid bone, also known as mastoid air cells, that develops in less than 30 days, usually as a complication of an infection of the middle ear, also known as otitis media [1]. Various bacterial species have been known to cause mastoiditis in both children and adults alike. The majority of isolates are generally *Streptococcus pneumoniae* and *Streptococcus pyogenes*, and other less common bacterial species such as *Haemophilus influenzae*, *Pseudomonas aeruginosa*, coagulase-negative *Staphylococcus*, etc. are also potential bacterium [2]. Most cases of acute mastoiditis occur in children younger than two years old [3]. In fact, one study which looked at the number of adults (>17 years old) with acute otitis media and acute mastoiditis at a tertiary center for a span of nine years had reported a small sample size of approximately 160 patients [4]. Therefore, it can be concluded that acute mastoiditis and other infections presenting as a complication of acute otitis media are quite rare, with an incidence of less than 0.5% [5]. This is primarily due to the use of antibiotics, which has resulted in a significant decrease in the overall incidence of mastoiditis [6]. However, this does not mean that when mastoiditis does occur, it should be neglected. In fact, prior studies have noted that when patients, particularly adults, develop acute mastoiditis, the disease can be quite aggressive. One case has documented a patient with severe mastoiditis that resulted in aphasia and altered mental status [7]. In rare instances, acute mastoiditis has also been shown to cause cerebral venous sinus thrombosis, due to the proximity of the mastoid air cells and the sigmoid sinuses. However, the majority of studies have only shown it to cause such a complication in children [8,9]. In even more rare situations, acute mastoiditis has also been shown to cause cerebral venous sinus thrombosis in adults. A recent case reported such an occurrence, but the patient was noted to have underlying thrombophilia, most likely secondary to her triple-negative breast cancer [10]. As such, we would like to present as well as discuss our case of a patient with cerebral sinus venous thrombosis secondary to acute mastoiditis without any significant underlying thrombophilia.

## Case presentation

The patient is a 55-year-old woman who presented with complaints of an 8/10 headache associated with photophobia and phonophobia, but no aura. She notes that the headache is more prominent on the right side of her head, originating from the periorbital region that radiates to the back of the ipsilateral-sided ear associated with blurry vision. The patient also expressed what she described as a "washing machine" in her ears, as well as intermittent left-hand numbness for the past few days. Her past medical history includes hypertension, type 2 diabetes mellitus, chronic venous insufficiency, and obesity with a body mass index of 46.8.

The physical exam showed that blood pressure was mildly hypertensive with systolic in the 140s and diastolic in the 80s; all other vital signs were unremarkable. The patient conveyed tenderness to palpation over the right temporal region. No occipital or post- or pre-auricular lymphadenopathy was appreciated. The bilateral external ears as well as the ear canals showed no lesions or erythema. The bilateral tympanic membranes showed no bulging, erythema, or effusions. The rest of the physical exam was unremarkable, except for lymphedema and stasis dermatitis of the right lower extremity from the ankle to below the knee.

Her initial tests showed an elevated C-reactive protein (CRP) as well as microcytic anemia based on the patient's low mean corpuscular volume (MCV) and hemoglobin. A complete blood count (CBC) showed a normal white blood cell count; however, she had bandemia and thrombocytopenia (Table 1). The blood smear also depicted abnormal shapes and sizes of her erythrocytes (Table 2).

**Table 1:** Initial blood work mmol/L: millimole/liter; mg/dL: milligrams/deciliter; units/L: units/liter; mg/dL: milligrams/deciliter; mm/hr: millimeters/hour; g/dL: grams/deciliter; fL: femtoliters; /mcL: cell/microliter; CRP: C-reactive protein; WBC: white blood cell; Hgb: hemoglobin; MCV: mean corpuscular volume

Lab value	Value	Reference
Sodium	134 mmol/L	136-145 mmol/L
Potassium	3.6 mmol/L	3.5-5.1 mmol/L
Chloride	99 mmol/L	98-107 mmol/L
Calcium	9.5 mg/dL	8.5-10.1 mg/dL
Magnesium	2.5 mg/dL	1.8-2.4 mg/dL
Phosphorus	3.4 mg/dL	2.5-4.9 mg/dL
Alanine transaminase	26 units/L	13-61 units/L
Aspartate transaminase	70 units/L	15-37 units/L
Direct bilirubin	0.3 mg/dL	0-0.2 mg/dL
Total bilirubin	0.8 mg/dL	0-1 mg/dL
Erythrocyte sedimentation rate	22 mm/hr	<20 mm/hr
CRP	6.67 mg/dL	<0.3 mg/dL
WBC count	5.2×10^3^/mcL	4.5-11×10^3^/mcL
Hgb	10.0 g/dL	13.2-17.4 g/dL
MCV	75.4 fL	80-98 fL
Platelet	71×10^3^/mcL	150-450x10^3^/mcL
Neutrophil %	54%	50-75%
Lymphocyte %	5%	20-45%
Bands %	45%	<12%
Monocyte %	14%	2-12%
Eosinophil %	2%	<6%
Absolute neutrophil	4.1×10^3^/mcL	1.8-7.7×10^3^/mcL
Absolute lymphocyte	0.3×10^3^/mcL	1.2-4.5×10^3^/mcL
Absolute monocyte	0.7×10^3^/mcL	0.1-1×10^3^/mcL
Absolute eosinophil	0.1×10^3^/mcL	<0.7×10^3^/mcL

**Table 2:** Cellular morphology

Cell morphology	Value
Smudge cells	+2
Large platelets	+3
Giant platelets	Occasional
Anisocytosis	+2
Microcytosis	+1
Hypochromia	+1
Polychromasia	+1
Teardrop cells	+1
Helmet cells	+1 (abnormal)
Elliptocytosis	+3
Burr cells	+1
Acanthocytosis	Occasional
Micro-poikilocytosis	Occasional
Blister cells	Occasional
Schistocytosis	Occasional

**Figure 1:**
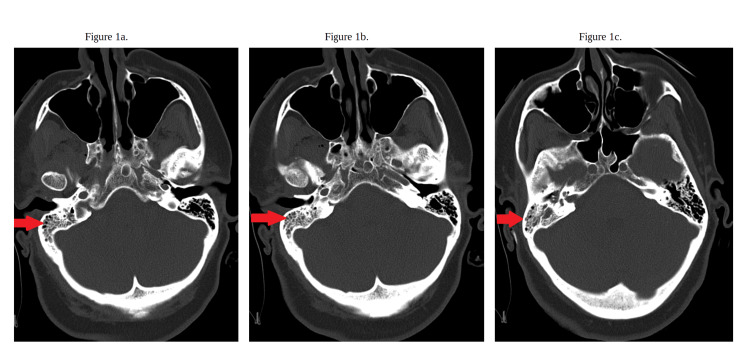
CT scan of the brain and head without contrast (a) The red arrow indicates right-sided mastoiditis at one slice of the CT scan. (b) The red arrow indicates right-sided mastoiditis at another slice of the CT scan. (c) The red arrow indicates right-sided mastoiditis at another slice of the CT scan. CT: computed tomography

**Figure 2:**
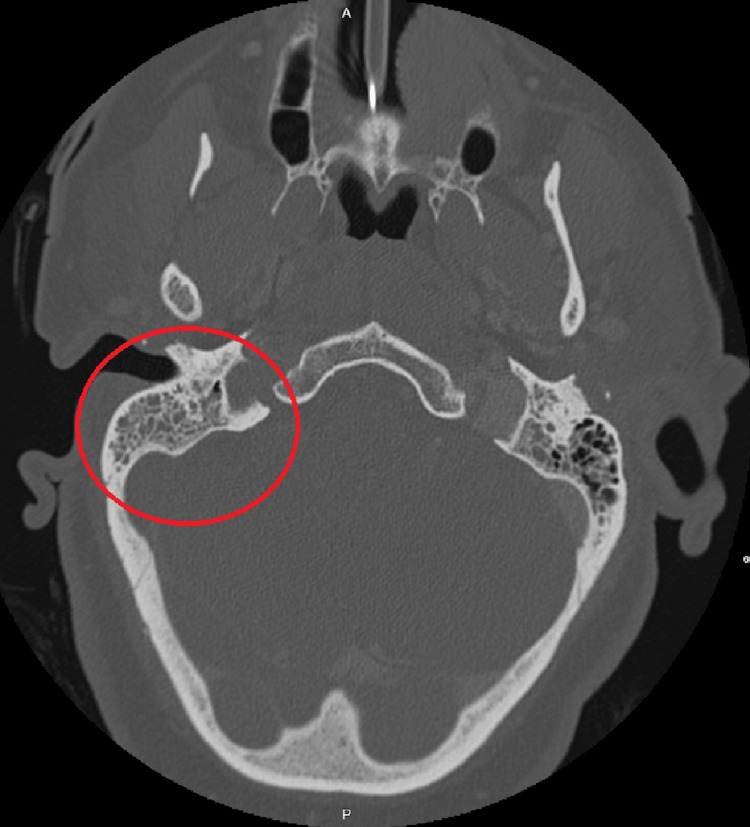
CT scan of the orbit and sella with contrast Opacification of the mastoid cells is present on the right side and is further highlighted by the red circle. The left mastoid bone is unremarkable without demonstrable middle ear disease or bone destruction. CT: computed tomography

Based on the patient's findings, she was admitted for concerns for giant cell arteritis and was started on prednisone 60 mg; however, she had no significant relief. A computed tomography (CT) scan of the brain and head without contrast showed right mastoiditis (Figure 1a-1c). No other acute intracranial findings were noted, and there was no evidence of any acute large vessel infarct. A CT of the orbit and sella with contrast was done to obtain further details of the patient's right mastoiditis (Figure 2). Due to clinical suspicion of giant cell arteritis, prednisone was continued, but the patient was started on 2 grams of ampicillin with 1 gram of sulbactam for right-sided mastoiditis, while a temporal artery biopsy was pending. The following day, the patient had developed bilateral upper extremity shaking as well as left-side facial and left upper extremity weakness. She was diagnosed with status epilepticus and was given lorazepam 4 mg twice. The patient was transferred to the intensive care unit with 24-hour electroencephalogram (EEG) monitoring. She was intubated and placed on mechanical ventilation with sedation because of her poor mentation, causing concerns about airway protection. The temporal artery biopsy was postponed because of the patient's new clinical picture.

**Figure 3:**
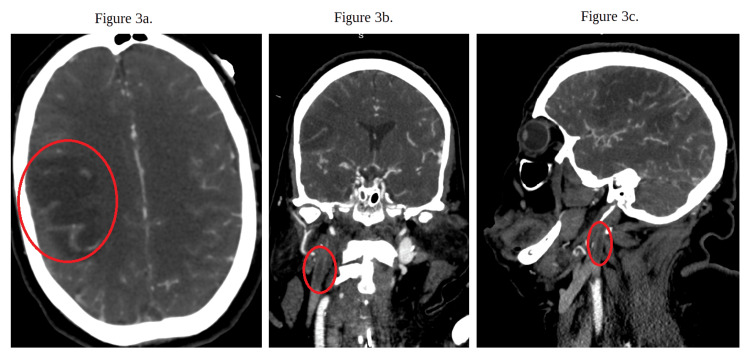
CT angiography of the brain and neck with contrast (a) The red circle highlights cortical infarcts. (b) This figure is a coronal view of the CT angiography of the brain and neck with contrast. The red circle shows the full occlusion due to a thrombosis of the upper right internal jugular vein. (c) This figure is a sagittal view of the CT angiography of the brain and neck with contrast. The red circle shows the full occlusion due to a thrombosis of the upper right internal jugular vein. CT: computed tomography

**Figure 4:**
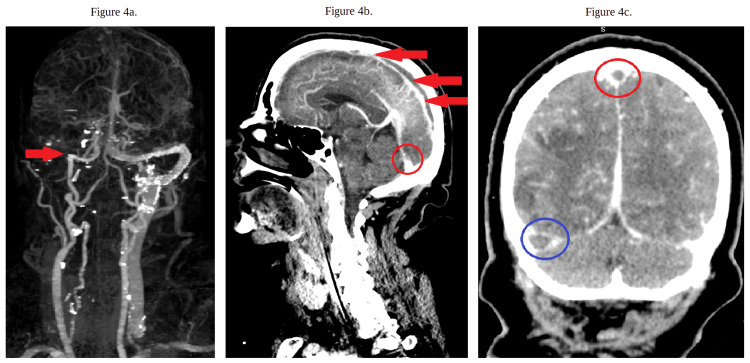
CT angiography of the brain and neck with contrast (a) The red arrow indicates a lack of flow. (b) The red circle highlights thrombosis of the confluence of sinuses, also known as the torcula. The red arrows also show superior sagittal sinus thrombosis. (c) The red circle indicates a filling triangular defect which portrays the empty delta sign which depicts dural venous sinus thrombosis of the superior sagittal sinus. The blue circle shows thrombosis of the right transverse and right sigmoid sinus. CT: computed tomography

A CT of the brain and head without contrast was repeated due to concerns of stroke. The results showed multiple acute infarcts in the right frontal, parietal, occipital, and temporal lobes with dural venous sinus thrombosis, which was new compared to her prior CT scan. A CT angiogram of the brain and neck with contrast was done to obtain further details regarding the patient's dural venous sinus thrombosis involving the superior sagittal sinus, confluence of sinuses, right transverse sinus, right sigmoid sinus, and upper right internal jugular vein (Figures 3a-4c).

**Figure 5:**
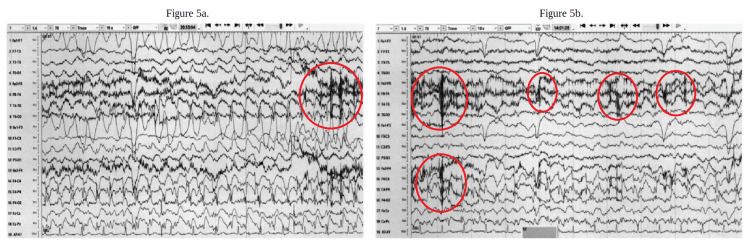
EEG (a) The EEG onset was at the right ventral region with sharp waves noted throughout the entire EEG but also more prominently depicted by the red circles. There is also a quick overriding activity for about 45 seconds to two minutes and 30 seconds, showing the seizures to be continuous, slow, and generalized. (b) This EEG onset was at the right central region with the duration of the seizures lasting less than one minute, showing the seizures to be continuous, slow, and generalized. There are also sharp waves noted throughout the entire EEG but also more prominently depicted by the red circles. EEG: electroencephalogram

Magnetic resonance imaging (MRI) was unable to be done due to the patient's body habitus. The EEG showed approximately seven focal as well as bilateral tonic-clonic seizures originating from the right central vertex (Figure 5a-5b). The patient was started on levetiracetam 2 grams intravenously twice daily, which resolved her seizures. Of note, the elevated levetiracetam dosing was decided based on the patient's weight and clinical picture. Neurological interventional radiology recommended a heparin drip for the thrombosis, and prednisone was stopped. Hematology/oncology was consulted for concerns about a hypercoagulable state, and otolaryngology, as well as infectious disease, was consulted for the patient's mastoiditis.

Hematology/oncology recommended a hypercoagulable workup (Table 3), and otolaryngology as well as infectious disease recommended continuing ampicillin 2 grams with sulbactam 1 gram with no surgical intervention. Despite being on treatment, the patient showed no significant neurologic improvement after one week, and the family decided to make the goals of comfort care only.

**Table 3:** Hypercoagulable panel Units/mL: units/milliliters; mcmol/L: micromoles/liter; IgA: immunoglobulin A; IgG: immunoglobulin G; IgM: immunoglobulin M; PTT-LA: partial thromboplastin time-lupus anticoagulant; DRVVT: dilute Russell's viper venom time; PS/PT Ab: phosphatidylserine/prothrombin antibody

Lab value	Value	Reference
Antithrombin III activity	<2 units/mL	<2 units/mL
B2 glycoprotein IgA	<2 units/mL	<2 units/mL
B2 glycoprotein IgG	<2 units/mL	<2 units/mL
B2 glycoprotein IgM	<2 units/mL	<2 units/mL
Cardiolipin Ab IgA	<2 units/mL	<2 units/mL
Cardiolipin Ab IgG	<2 units/mL	<2 units/mL
Cardiolipin Ab IgM	<2 units/mL	<2 units/mL
Factor V Leiden	Negative	Negative
Homocysteine	6.9 mcmol/L	<10.4 mcmol/L
Lupus anticoagulant	Not detected	Not detected
PTT-LA	127 seconds	<40 seconds
DRVVT screen	42 seconds	<45 seconds
Hexagonal phase confirm	Positive	Negative
PS/PT Ab (IgM)	<9 units	30 units
PS/PT Ab (IgG)	<9 units	30 units
Antiphospholipid antibody	Negative	Negative
Protein C	95%	70-180%
Protein S	47%	60-140%
Prothrombin factor II mutation	Negative	Negative
Thrombin clotting time	>100 seconds	13-19 seconds

## Discussion

Cerebral venous thrombosis is a rare complication of acute mastoiditis and occurs primarily in the pediatric population [8,9]. However, there are some instances where such complications occur in adults. In the adult population, the incidence of cerebral venous thrombosis is one to two cases per 100,000, and it is three times more prevalent in women than men [11,12]. This is believed to be due to hypercoagulable states that women are more prone to due to pregnancy and oral contraceptives [13]. However, other risk factors have been attributed to the cause of cerebral venous thrombosis, and like in other thrombotic diseases, such risk factors are generally associated with stasis, hypercoagulability, and endothelial wall damage [14,15]. Many commonly acquired risk factors can vary from cancer, obesity, infections, medications, pregnancy, etc. [10-15] in addition to inherited genetic prothrombotic conditions such as factor V Leiden, protein C and S deficiency, etc. [14].

Studies have shown that patients with cerebral venous thrombosis tend to depict a wide range of signs. About 90% of patients develop severe headaches that can progress to severe debilitating symptoms. In fact, 92.2% of patients develop neurological diseases such as seizures, altered mental status, coma/stupor, and/or aphasia. Other clinical manifestations such as ophthalmological diseases also occurred, with an estimated 55% of patients developing papilledema, diplopia, and/or visual deficits [14,16]. In patients with septic cerebral venous thrombosis, symptoms such as fevers and orbital cellulitis can also occur [14]. As a result, these clinical manifestations can vary heavily and their timing as well, with occurrences reported from two days to six months [14]. Patients who have this complication mainly develop neurological symptoms, and radiological imaging is a key diagnostic tool used to make a diagnosis. Magnetic resonance venography and CT venography have been shown to be crucial imaging modalities for confirmation with a sensitivity of approximately 95% [17]. However, in some instances where such imaging modalities are unavailable, both CT angiography and magnetic resonance angiography are recommended [14]. Once the diagnosis is confirmed, administering anticoagulation, particularly heparin (either unfractionated or low molecular weight), should be immediate in order to restore flow to the occluded venous vessels. Such treatment should be started promptly to prevent further complications.

Due to the contiguous structure of the cerebral venous sinuses, localized infected areas can allow the development of complications around the cerebral venous sinuses [13]. In this case, inflammation caused by mastoiditis resulted in a prothrombotic state, causing cerebral venous thrombosis. Yet, infections of the middle ear resulting in cerebral venous sinus thrombosis tend to be rare due to the advancement of antibiotics [14]. In prior cases, patients who have developed cerebral venous thrombosis tend to have prothrombotic risk factors, and although this patient did have co-morbidities such as type 2 diabetes mellitus, hypertension, chronic venous insufficiency, and increased body mass index, such risk factors were less associated with cerebral venous thrombosis [14]. The fact that the patient also had a negative thrombophilia workup (Table 3) also further supports a unique case in which the patient developed a hypercoagulable state secondary to mastoiditis resulting in cerebral venous thrombosis without any other recognizable prothrombotic conditions. She did in fact have a positive hexagonal phase confirm, which indicated the presence of lupus anticoagulant by measuring activated partial thromboplastin time (aPTT) [18]. However, the hypercoagulable panel was done after the patient was started on heparin because an immediate response was required once imaging had confirmed cerebral venous thrombosis. This, as a result, would lead to a false positive. This is also supported by the fact that the lupus anticoagulant was directly tested and was noted to be negative during the workup (Table 3). Although companies do add a neutralizer to prevent false positives due to heparin, such neutralizers are not sufficient in the high doses of heparin from which this patient was receiving [18].

## Conclusions

Cerebral vein thrombosis is a very rare complication of acute mastoiditis, particularly in adults. Most cases have been associated with a hypercoagulable condition. In our patient, such a condition was not found. A high level of suspicion is required to consider this uncommon but highly lethal diagnosis. In patients with concerns for neurological symptoms alongside a consistent clinical picture, prompt imaging should be done to rule out cerebral vein thrombosis. Upon diagnosis, immediate treatment with heparin should be started to attempt to restore venous flow. Further workup should be done to ensure to look for any other condition that can cause a hypercoagulable state, since such conditions should be addressed to prevent further thrombotic events. Unfortunately, even patients without common prothrombotic states can still develop this severe complication, which carries a very high morbidity and mortality.
